# A Novel Circular RNA circSPEF2 Regulates Testis Development in Crucian Carp

**DOI:** 10.3390/biology15090669

**Published:** 2026-04-23

**Authors:** Fang Gou, Yanmei Gao, Rui Wang, Dongmei Zhong, Rong Yang, Shaojun Liu

**Affiliations:** 1 Engineering Research Center of Polyploid Fish Reproduction and Breeding of the State Education Ministry, College of Life Sciences, Hunan Normal University, Changsha 410081, China; 202470142961@hunnu.edu.cn (F.G.); 202320142793@hunnu.edu.cn (Y.G.); 202520143006@hunnu.edu.cn (R.W.); 202410140325@hunnu.edu.cn (D.Z.); 2 Yuelushan Laboratory, Changsha 410128, China

**Keywords:** circRNA, gonad development, RNA-seq, circSPEF2

## Abstract

Circular RNAs (circRNAs) exhibit significant sex- and development stage-specific expression patterns in the gonads of various fish species, yet their functions and regulatory mechanisms in male reproductive development remain largely unexplored in crucian carp (*Carassius auratus*). In this study, we characterized the expression features and biological functions of circSPEF2, a circular RNA derived from the reproduction-related gene *spef2*. Our results showed that circSPEF2 expression was markedly elevated in mature testes and progressively upregulated during gonadal maturation. Functional studies suggested that circSPEF2 likely does not act through a ceRNA-dependent mechanism. Transcriptome sequencing following circSPEF2 overexpression identified 45 upregulated and 70 downregulated differentially expressed genes, with GO and KEGG enrichment analyses revealing significant alterations in multiple gonadal development-related genes and signaling pathways. Subsequent siRNA-mediated knockdown of circSPEF2, combined with qRT-PCR validation, confirmed that circSPEF2 positively regulates the expression of genes associated with cell maturation and differentiation, including *prdm1a*, *lamc2*, and *slc25a27*, while concurrently suppressing that of proliferation- and apoptosis-related genes such as *wnt8b*, *cpeb3*, and *bcl2l11*. Furthermore, RNA pull-down combined with mass spectrometry identified three candidate circSPEF2-binding proteins, namely, hnRNP A/B, SRSF2, and CFAP263. Collectively, these findings indicate that circSPEF2 plays an important role in male gonadal development in fish and provide new insights into the post-transcriptional regulatory mechanisms underlying vertebrate male reproduction.

## Simple Summary

Circular RNAs exhibit distinct expression patterns in fish gonads, yet their specific functions during male gonadal development remain largely elusive. In this study, we identified a testis-enriched circular RNA, circSPEF2, in crucian carp. Through a combination of overexpression, RNA interference, and mass spectrometry-based proteomic analysis, we demonstrated that circSPEF2 promotes the expression of genes associated with cell maturation and differentiation, while suppressing the expression of genes related to proliferation and apoptosis. Furthermore, we identified three candidate RNA-binding proteins that interact with circSPEF2. Collectively, our findings reveal that circSPEF2 serves as a critical post-transcriptional regulator of male gonadal development in fish, offering novel insights into the molecular mechanisms underlying circRNA-mediated reproductive regulation in vertebrates.

## 1. Introduction

Freshwater aquaculture is an important source of animal protein worldwide, and reproductive performance is crucial for its sustainable development. Understanding the molecular regulatory mechanisms of gonadal development in bony fish is important for revealing fish reproductive biology and improving breeding efficiency. Crucian carp (*Carassius auratus*), an important freshwater fish in China and East Asia, has become a key model for studying gonadal development in teleosts because of its short reproductive cycle and the ease with which experimental materials can be obtained. Previous studies have shown that fish sex differentiation and gonadal development are regulated by a variety of conserved and species-specific genes, including *dmrt1*, *sox9*, *amh*, and *gsdf*, related to the male pathway, and *foxl2*, *cyp19a1a*, *wnt4*, and *rspo1*, related to the female pathway [1]. Notably, the *SPEF2* gene has been confirmed to be closely related to male sterility in mammals, suggesting that it may have conserved reproductive regulatory functions in different vertebrate taxa. Spontaneous mutations in *SPEF2* were first found to cause sperm infertility in Finnish Yorkshire boars [2,3]. This gene encodes sperm flagellar axonemal protein 2 (SPEF2), which is involved in the formation of sperm tail structures. Subsequent studies showed that the loss of *Spef2* function in mouse mutants results in a “primary ciliary dyskinesia (PCD)-like” phenotype, accompanied by severe spermatogenesis defects [4,5]. Later studies demonstrated that *Spef2* is involved in Golgi-associated transport in male germ cells [6]. Recent studies have increasingly shown that mutations in the *SPEF2* gene are responsible for multiple morphological abnormalities of the flagella (MMAF) in humans, a condition closely linked to male infertility [7,8,9]. Transcriptomic data indicate that *spef2* is highly expressed in the gonads of male fish, with negligible expression observed in females, and its expression levels in sterile triploid testes are significantly lower than those in fertile diploid testes. However, the specific function of the *spef2* gene in the development of fish gonads remains to be investigated [10,11].

circRNAs are covalently closed non-coding RNAs characterized by structural stability and tissue-specific expression, and they function through competing endogenous RNA (ceRNA) networks, protein interactions, and direct translation, thereby regulating diverse biological processes [12,13,14,15]. Extensive sequencing data indicate that circRNAs are specifically expressed in animal reproductive and developmental systems, likely playing crucial regulatory roles [16,17,18,19]. For instance, in mammals, circRNAs such as circRNA40370, derived from the *RICTOR* gene, and circRNA9244, originating from the *SMARCA5* gene, have been identified as biomarkers for assessing the developmental stages of porcine testes [20,21]. Similarly, studies in fish have shown that circDMRT1 regulates the expression of the *gsdf* gene by competitively binding to cse-miR-196 through a ceRNA mechanism, playing a critical role in sex determination and differentiation in tongue sole [22]. Additionally, circSND1 has been demonstrated to regulate *foxl2* expression during sex reversal in Japanese eel by acting as a sponge for mal-miR-135b/c [23]. However, research investigating the molecular mechanisms by which circRNAs influence gonadal development in crucian carp has yet to be conducted.

In this study, we identified circSPEF2 as a testis-enriched circRNA, with elevated expression in mature testes, and we systematically explored its functional relevance. Our findings revealed a strong positive correlation between circSPEF2 expression and progressive testicular development. Additionally, we found that circSPEF2 may regulate critical genes and signaling pathways involved in gonadal development through a mechanism independent of the ceRNA pathway. Importantly, we identified three candidate circSPEF2-interacting proteins, providing key clues for understanding the molecular regulation of circSPEF2 in crucian carp male gonadal development.

## 2. Materials and Methods

### 2.1. Experimental Materials

Fish were obtained from the China National Engineering Research Center for Polyploid Fish Breeding and Reproduction (Hunan Normal University) and maintained under standard conditions. Four fish groups, namely RCC (red crucian carp), WCC (white crucian carp), WR (WCC ♀ × RCC ♂), and WR-II (WR ♀ × WCC ♂), were included in the study, each consisting of three independent biological replicates using distinct individuals. The animal experiments in this study were approved by the Biomedical Research Ethics Committee of Hunan Normal University (Approval Number: 20260309). All experimental procedures were conducted in strict accordance with the ethical guidelines and animal welfare regulations of the committee.

### 2.2. Total RNA Extraction and Pcr Amplification

Grind tissue samples from RCC, WCC, WR, and WR-II in liquid nitrogen. Lyse them with AG RNAex Pro reagent (Accurate Biotechnology Co., Ltd., Changsha, China). Add and mix chloroform. Centrifuge the mixture to separate the aqueous and organic phases. Transfer the aqueous phase and mix with an equal volume of isopropanol. Incubate the tubes on ice. Collect RNA pellets by centrifugation, wash with ice-cold 80% ethanol, air-dry, and dissolve in DEPC-treated water to obtain total RNA. Only RNA samples meeting the following quality criteria were used for subsequent reverse transcription and PCR amplification: A260/A280 ratio of 1.8–2.0, A260/A230 ratio > 2.0, and concentration ≥ 300 ng/μL. Total RNA samples were reverse-transcribed using the HiScript II 1st Strand cDNA Synthesis Kit (+gDNA wiper) (Vazyme Biotech Co., Ltd., Nanjing, China), and PCR amplification was subsequently performed with Phanta Max Super-Fidelity DNA Polymerase (Vazyme Biotech Co., Ltd., Nanjing, China). The amplification products were analyzed by 2% agarose gel electrophoresis. All primer sequences are listed in Table S1.

### 2.3. Extraction of Genomic DNA

Genomic DNA (gDNA) was extracted from the caudal vein blood of sexually mature RCC using a TIANGEN TIANamp Genomic DNA Kit (TIANGEN Biotech, Beijing, China; cat. no. DP304). ACD anticoagulant (Sangon Biotech Co., Ltd., Shanghai, China) was added during blood collection. Following this, 200 μL of the anticoagulated whole blood was lysed with 200 μL of GA buffer and 20 μL of Proteinase K, and RNA was removed by adding 4 μL of RNase A (100 mg/mL). Subsequently, the DNA mixture was applied to the affinity purification column. Following sequential washing and elution, purified genomic DNA (gDNA) was obtained. The concentration of the purified gDNA was determined, and the sample was stored at −20 °C.

### 2.4. Quantitative Real-Time Polymerase Chain Reaction Analysis

Total RNA was extracted from tissue samples of testes, ovaries, brain, liver, kidney, spleen, intestine, and muscle from RCC. First-strand cDNA was synthesized from total RNA using HiScript II Q RT SuperMix for qPCR (+gDNA wiper) (Vazyme Biotech Co., Ltd., Nanjing, China) with random hexamer primers, and the resulting cDNA products were diluted 10-fold before subsequent analysis. Quantitative PCR was performed using 2× PowerUp SYBR Green Master Mix (Applied Biosystems, Foster City, CA, USA) according to the manufacturer’s instructions. β-actin was used as the internal control, and the relative gene expression levels were calculated using the 2^(−ΔΔCt) method. Specificity was confirmed by melting curve analysis and agarose gel electrophoresis. All reactions were run in triplicate with three biological replicates.

### 2.5. RNase R Digestion

RNase R digestion was performed to evaluate the stability of circSPEF2. RNA extracted from the gonadal tissues of the RCC, WCC, WR, and WR-II strains was divided into two groups: RNase+ (1 μg RNA, 2.5 U RNase R (BIOSEARCH Technologies, Petaluma, CA, USA), 1 μL 10× Reaction Buffer, RNase-free water to 10 μL) and an enzyme-free control (using RNase-free water instead of RNase R). Both reactions were incubated at 37 °C for 30 min and then terminated by heating at 70 °C for 10 min.

### 2.6. Fluorescence In Situ Hybridization

Fresh testicular tissues from male red crucian carp were fixed in RNase-free 4% paraformaldehyde for 1 h, processed into paraffin blocks, and sectioned at 5 µm. After two 8 min washes in fresh xylene (for a total of 16 min) to remove paraffin, the sections were rehydrated through a graded ethanol series and rinsed twice in absolute ethanol (5 min each). All subsequent steps were performed strictly according to the protocol provided by the FISH In Situ Hybridization Kit (Shanghai Gefan Biotechnology Co., Ltd., Shanghai, China). Following probe hybridization and stringency washes, the slides were mounted with DAPI-containing antifade medium, coverslipped, sealed with clear nail polish, and examined under a fluorescence microscope for imaging.

### 2.7. Dual-Luciferase Reporter

MiRNA mimics targeting the predicted sites on circSPEF2 were designed. Two luciferase reporter vectors, circSPEF2-wt and circSPEF2-mut, were constructed. HEK293T cells, cultured in high-glucose DMEM (CellorLab Co., Ltd., Wuhan, China) with 10% FBS (Umedium Co., Ltd., Wuhan, China), penicillin, and streptomycin at 37 °C and 5% CO_2_, were seeded in a 6-well plate at a density of 10^4^ cells per well. After 24 h, the cells were co-transfected with 500 ng of the reporter plasmid (circSPEF2-wt or circSPEF2-mut) and 100 nM of miRNA mimics (cpo-miR-143-3p_R+1 and mmu-let-7i-5p) or a negative control, using Lipofectamine 3000 (Invitrogen Co., Ltd., Carlsbad, CA, USA). Forty-eight hours post-transfection, luciferase activity was measured using the Dual-Luciferase^®^ Reporter Assay System (Promega Co., Ltd., Madison, WI, USA). Values were normalized, and fold changes were calculated.

### 2.8. EPC Cell Culture

EPC (Epithelioma Papulosum Cyprini) cells were cultured in DMEM (CellorLab Co., Ltd., Wuhan, China) medium containing 10% fetal bovine serum (Umedium Co., Ltd., Wuhan, China), 100 U mL^−1^ of penicillin, and 100 µg mL^−1^ of streptomycin at 28 °C and 5% CO_2_. The culture medium was replaced at 2–3 day intervals. Once cells reached 80–90% confluence, they were digested with 0.25% trypsin solution (containing EDTA and phenol red, CellorLab Co., Ltd., Wuhan, China) and subcultured. After resuscitation, the cells were passaged twice, and subsequent experiments were conducted once stable growth was achieved.

### 2.9. Overexpression and Knockdown of circSPEF2

EPC cells with a favorable growth status were inoculated into 6-well plates at a density of 5 × 10^5^ cells/well. Transfection was initiated once cell confluence reached 40–50%. The circSPEF2 overexpression plasmid was transfected into EPC cells using a liposome-based method. After 6 h, the culture medium was replaced, and the cells were further cultured for 24 h. Subsequently, qRT-PCR was performed to confirm that circSPEF2 expression had increased by more than 3-fold and that cell viability had exceeded 95%, after which the cells were directly used for subsequent experiments. For specific knockdown of circSPEF2, EPC cells that had been transfected with the circSPEF2 overexpression plasmid for 24 h were further transfected with siRNA using the liposome-based method. Transfected cells were incubated for 6 h, after which the medium was replaced with fresh medium and the culture was prolonged for 48 h. Thereafter, cells were collected, and total RNA was isolated for subsequent analyses.

### 2.10. RNA-Seq and Differential Gene Expression Analysis in EPC Cells Following CircSPEF2 Overexpression

The EPC cells transfected with pcDNA3.1 and oe-circSPEF2 plasmids were analyzed via mRNA sequencing. Total RNA was extracted from both groups of EPC cells using AG RNAex Pro reagent (Accurate Biotechnology Co., Ltd., Changsha, China), with each group comprising three biological replicates. Total RNA samples were purified, and library construction and sequencing were performed by Shanghai Meiyi Biopharmaceutical Biotechnology Co., Ltd. (Shanghai, China). The mRNA-seq library was prepared using the Illumina^®^ Stranded mRNA Prep, Ligation kit (Illumina, Inc., San Diego, CA, USA). Briefly, mRNA was enriched from total RNA using oligo(dT) magnetic beads, followed by fragmentation, double-stranded cDNA synthesis, end repair, adapter ligation, magnetic bead-based size selection, and PCR amplification. Sequencing was conducted on the NovaSeq X Plus platform (PE150). After preprocessing, the raw reads were aligned to the reference genome using HISAT2 (version 2.1.0), assembled with StringTie (version 2.1.2), and gene expression levels were quantified using RSEM (version 1.3.3) as TPM values. Statistical identification of differentially expressed genes (DEGs) was carried out with the DESeq2 package (version 1.10.1). Genes exhibiting |log_2_FC| ≥ 1 and adjusted *p*-value (Benjamini–Hochberg FDR) < 0.05 were regarded as significantly differentially expressed. Gene Ontology (GO) enrichment analysis was performed using Goatools software (version 0.6.5), whereas Kyoto Encyclopedia of Genes and Genomes (KEGG) pathway enrichment analysis was carried out with the Python scipy package (version 1.10.1). Significantly enriched terms or pathways were identified based on an adjusted *p*-value (Bonferroni) < 0.05 with the whole-transcriptome set as the background.

### 2.11. RNA Pull-Down and Mass Spectrometry

Total proteins were extracted from immature and mature male gonadal tissues of RCC. The biotin-labeled circSPEF2 probe was incubated with streptavidin magnetic beads (Thermo Fisher Scientific Inc., Waltham, MA, USA) to prepare probe–bead complexes, which were then incubated with equal amounts of total protein from immature and mature gonadal tissues at 4 °C overnight. The pulled-down proteins were separated via SDS-PAGE and visualized with silver staining using a Fast Silver Stain Kit (Beyotime Biotechnology Co., Ltd., Shanghai, China; cat. no. P0017S) according to the manufacturer’s instructions. Based on the silver staining results, differential protein bands within the 35–55 kDa molecular weight range were excised. The gel pieces were destained, reduced, alkylated, and digested with trypsin at 37 °C overnight. The peptide samples were desalted on a nanoViper C18 trap column, then subjected to liquid chromatography-tandem mass spectrometry (LC-MS/MS) analysis using an UltiMate 3000 RSLC nano system (Thermo Fisher Scientific Inc., Waltham, MA, USA) coupled with a Q Exactive Plus mass spectrometer (Thermo Fisher Scientific Inc., Waltham, MA, USA). The acquired raw data were processed and searched against the target protein database via PEAKS Studio 12 software. Mass spectrometry data were filtered with the quality control criteria of −10LgP ≥ 20, Unique ≥ 1, Peptides ≥ 2, and Coverage ≥ 5%, thereby eliminating low-confidence identifications.

### 2.12. Statistical Analysis

Fold changes in target gene expression were calculated using the 2−ΔΔCT method. The stability of the Ct values of the housekeeping gene (β-actin) was assessed using the GeNorm algorithm (M value < 0.5). Data are presented as the mean ± standard deviation (mean ± SD). A one-way ANOVA and a two-way ANOVA (GraphPad Prism 10.0) were applied, with *p* < 0.05 considered significant.

## 3. Results

### 3.1. Identification of circSPEF2 in Crucian Carp

**Figure 1:**
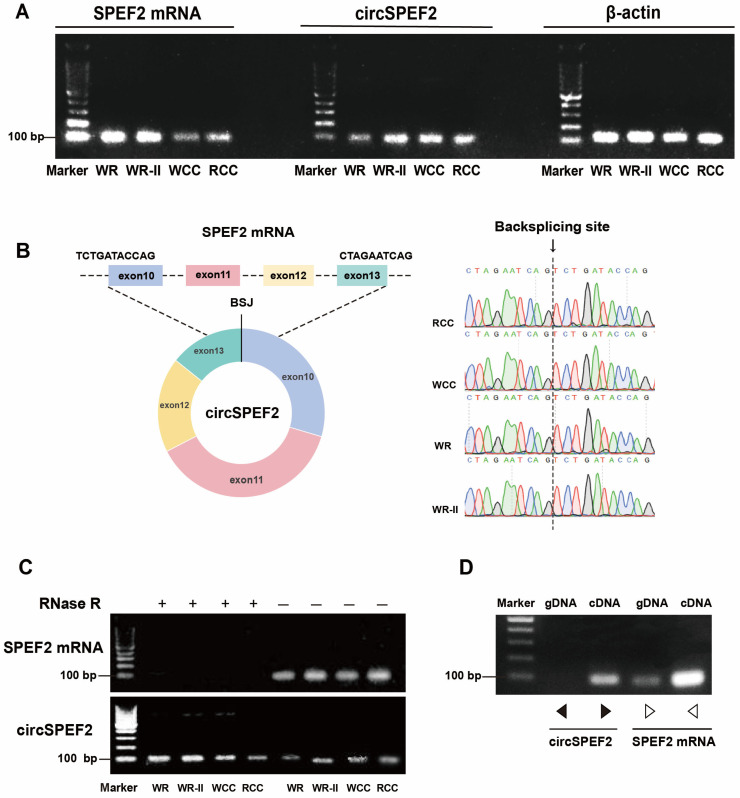
Identification of circSPEF2 in crucian carp. (**A**) Target PCR products were analyzed using agarose gel electrophoresis following the amplification of circSPEF2 and linear *spef2* mRNA in RCC, WCC, WR, and WR-II. (**B**) Sanger sequencing confirmed the presence of the same reverse splice site in the circSPEF2 RT-PCR products across all four crucian carp species. (**C**) RT-PCR and agarose gel electrophoresis were conducted to evaluate the expression of circSPEF2 and linear *spef2* mRNA in the presence or absence of RNase R. (**D**) RT-PCR confirmed the existence of circSPEF2 and its circular structure in RCC. This was specifically amplified only in cDNA using divergent primers and not in gDNA. The black and white arrows in the figure indicate the positions of the divergent and convergent primers, respectively.

RCC, WCC, and their hybrid progenies WR and WR-II exhibit stable and conserved genetic backgrounds, thus providing a reliable foundation for the subsequent experimental studies [24,25]. To investigate the regulatory roles and underlying molecular mechanisms of circRNAs in the testicular development of crucian carp, we performed a comparative transcriptome analysis using RNA-seq on immature and mature RCC testis tissues and identified a key candidate circRNA, namely, circSPEF2 (ID: circRNA3213). Its parental gene, *spef2*, encodes sperm flagellar protein 2, which is primarily localized to the flagellar axoneme and plays a critical role in sperm motility and orientation. Numerous studies have demonstrated that mutations or abnormal expression of *spef2* can lead to male infertility or other reproductive disorders [2,3]. A genomic analysis revealed that circSPEF2 is composed of exons 10, 11, 12, and 13 of the *spef2* gene. We designed convergent and divergent primers using RCC as the reference sequence. PCR amplification was performed in RCC, WCC, and their hybrid progenies WR and WR-II (Figure 1A), and the RT-PCR products of circSPEF2 were sequenced using Sanger methods. This confirmed that circSPEF2 in all four crucian carp species exhibited the same reverse splicing sites specific to circRNAs (Figure 1B). These results indicate that circSPEF2 expression is significantly conserved across different genetic backgrounds of *Carassius*, laying the foundation for its potential use as a universal molecular marker for the regulation of crucian carp gonadal development. The stability of circRNA was confirmed through RNase R digestion experiments, which showed that circSPEF2 remained intact in four strains of crucian carp after RNase R treatment, while linear *spef2* mRNA was completely degraded (Figure 1C). Given the conserved expression of circSPEF2 across the four crucian carp genotypes, parallel PCR using the same primers on RCC gDNA and testicular cDNA was conducted. Agarose gel electrophoresis revealed specific bands only in the cDNA template, confirming that circSPEF2 is a circular transcript formed by exon reverse splicing (Figure 1D). Collectively, these experiments confirm that circSPEF2 is a genuine circRNA, identifying it as a primary candidate for investigating the mechanisms of circRNAs in the testicular development of crucian carp.

### 3.2. Characterization of circSPEF2 in RCC

**Figure 2:**
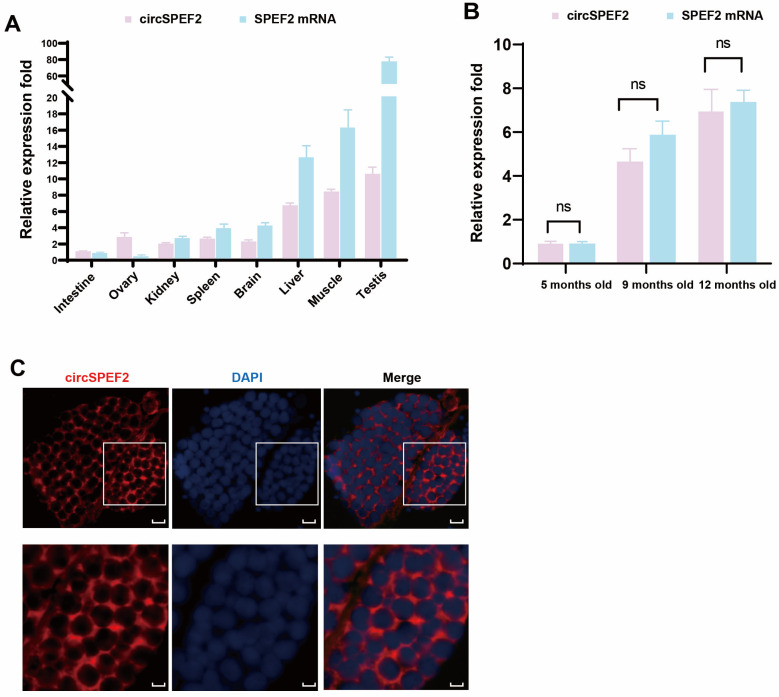
Characterization of circSPEF2 in RCC. (**A**) QRT-PCR was performed to measure the relative abundance of circSPEF2 and linear *spef2* mRNA in the intestines, ovaries, kidneys, spleens, brains, livers, muscles, and testes of RCC. (**B**) QRT-PCR was conducted to assess the relative expression of circSPEF2 and linear *spef2* mRNA at different developmental stages of RCC testes: 5 months old (immature), 9 months old (mid-development), and 12 months old (full maturity). ns, not significant. (**C**) Fluorescence in situ hybridization was utilized to analyze the subcellular localization of circSPEF2 in the testes of 12-month-old RCC. circSPEF2 is shown in red, and nuclei are counterstained with DAPI (blue). The scale bars for the upper and lower rows are 20 and 5 μm, respectively.

To assess the tissue distribution of circSPEF2 in crucian carp, we measured its relative abundance in eight tissues, namely, the testes, ovaries, brain, liver, kidneys, spleen, intestines, and muscles, using RCC as the model. QRT-PCR analysis revealed that circSPEF2 and *spef2* mRNA were highly expressed in the testes. This suggests that the formation and function of circSPEF2 are primarily concentrated in the male reproductive system, providing evidence for its potential role in regulating testicular development (Figure 2A). We subsequently collected testes from RCC at three developmental stages, namely, 5 months old (immature), 9 months old (mid-development), and 12 months old (fully mature), and performed a time series analysis via qRT-PCR. The results indicated that the relative expression levels of circSPEF2 and *spef2* mRNA increased monotonically with testicular development, suggesting that circSPEF2 may be involved in spermatogenesis and testicular maturation through mechanisms such as competitive miRNA adsorption or as a regulatory factor (Figure 2B). To clarify the site of action for circSPEF2, we conducted fluorescence in situ hybridization (FISH) on the testes of 12-month-old RCC using a specifically labeled probe. The results revealed that the circSPEF2 signal was primarily enriched in the cytoplasm, with minimal fluorescence observed in the nucleus. This indicates that the cytoplasm serves as the main functional platform for circSPEF2, establishing a subcellular localization basis for its role as an miRNA sponge or translation regulatory scaffold (Figure 2C).

### 3.3. circSPEF2 Does Not Exhibit the CeRNA Effect in RCC

**Figure 3:**
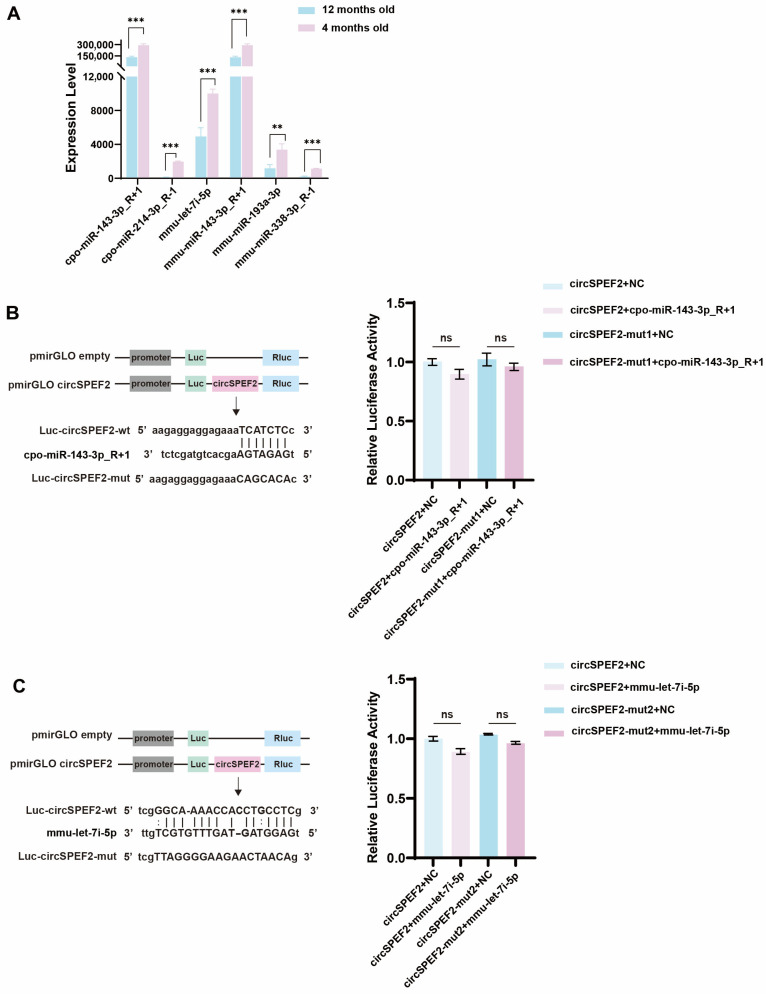
CircSPEF2 does not exhibit the ceRNA effect. (**A**) The five top-ranked miRNAs predicted to interact with circSPEF2 in RCC testes. (**B**) The left panel shows a schematic illustration of the constructed luciferase reporter vectors of circSPEF2-wt and circSPEF2-mut; the right panel illustrates the regulatory relationship between cpo-miR-143-3p_R+1 and circSPEF2, which was measured in HEK293T cells using dual-luciferase reporter assays. (**C**) The left panel shows a schematic illustration of the constructed luciferase reporter vectors of circSPEF2-wt and circSPEF2-mut; the right panel illustrates the regulatory relationship between mmu-let-7i-5p and circSPEF2, which was measured in HEK293T cells using dual-luciferase reporter assays. ** *p* < 0.01, *** *p* < 0.001; ns, not significant.

To identify potential interacting miRNAs for circSPEF2 in the testes of red crucian carp, we combined the miRNAda and miRanda algorithms to predict five high-confidence target miRNAs. Among these, the top three in expression levels—cpo-miR-143-3p_R+1, mmu-miR-143-3p_R+1, and mmu-let-7i-5p—exhibited a significant downregulation trend in red crucian carp testes from 4 to 12 months of age. This expression change showed a significant negative correlation with the continuously upregulated expression of circSPEF2 during testis maturation, consistent with the characteristics typical of ceRNA interactions. Notably, cpo-miR-143-3p_R+1 (derived from red crucian carp) has identical sequences to mmu-miR-143-3p_R+1 (derived from mice). Given this high sequence conservation, we selected cpo-miR-143-3p_R+1 and mmu-let-7i-5p for subsequent validation using the dual-luciferase reporter gene assay (Figure 3A). We constructed two luciferase reporter vectors, circSPEF2-wt and circSPEF2-mut, by introducing point mutations at the predicted cpo-miR-143-3p_R+1 binding site within circSPEF2 (Figure 3B, left). The vectors were co-transfected with cpo-miR-143-3p_R+1 mimics or negative controls into HEK293T cells. Cells were collected at 48 h for luciferase activity detection. The results indicated that in the co-transfection group of circSPEF2-WT and cpo-miR-143-3p_R+1, luciferase activity did not significantly decrease compared with that in the other treatment groups (Figure 3B, right). This suggests that circSPEF2 may not function as an adsorber for cpo-miR-143-3p_R+1. Using the same experimental strategy, we further assessed the interaction between mmu-let-7i-5p and circSPEF2, which also showed no significant changes in luciferase activity (Figure 3C). This indicates that circSPEF2 does not act as a molecular sponge for mmu-let-7i-5p. In summary, our validation using the dual-luciferase reporter system suggests that circSPEF2 is unlikely to participate in regulation via the ceRNA mechanism. Future studies will focus on exploring other potential biological functions of circSPEF2.

### 3.4. Sequencing Results After Overexpression of circSPEF2

**Figure 4:**
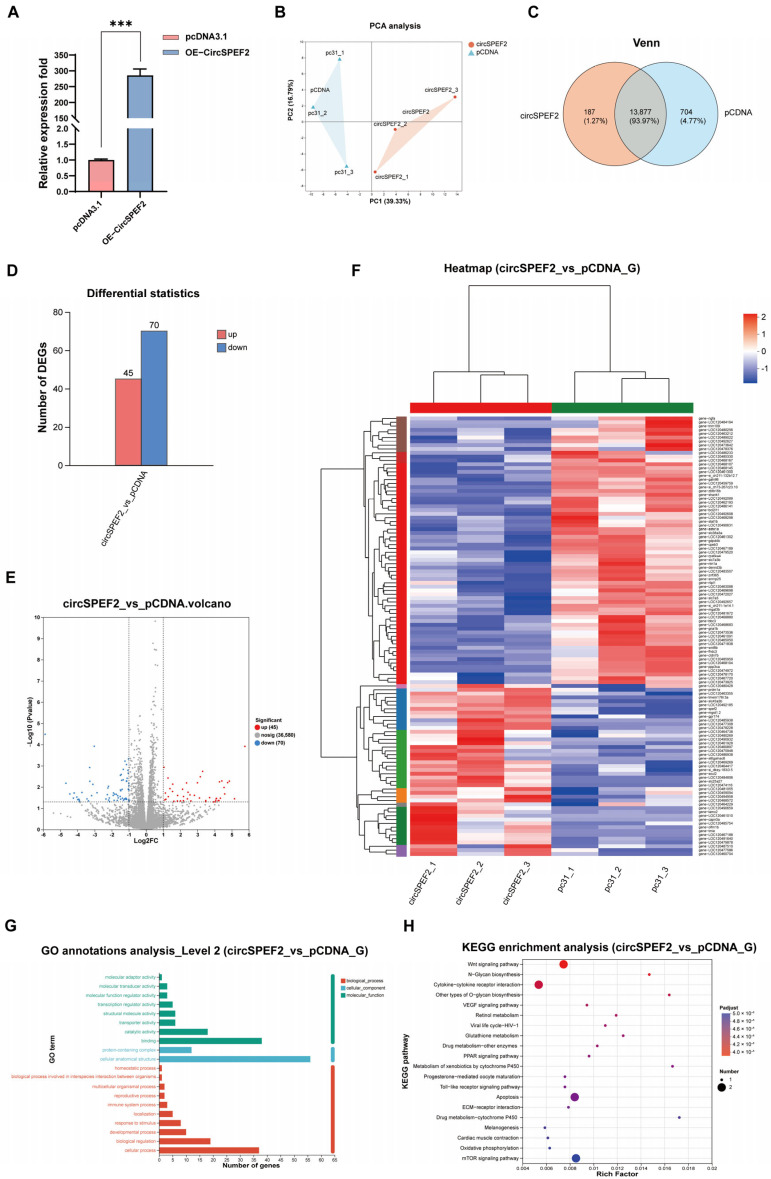
RNA-Seq results following overexpression of circSPEF2. (**A**) Overexpression of circSPEF2 in EPC cells was validated via qRT-PCR. *** *p* < 0.001. (**B**) Principal component analysis (PCA) of samples. (**C**) Venn diagram of co-expressed genes between oe-circSPEF2 and pcDNA3.1. (**D**) The number of DEGs in the oe-circSPEF2 group versus the pcDNA3.1 control. (**E**) Volcano plot of DEGs. (**F**) Heatmap of DEG expression patterns. (**G**) GO enrichment analysis of DEGs (biological process, cellular component, and molecular function). (**H**) KEGG pathway enrichment analysis of DEGs.

To further investigate the potential function of circSPEF2, we constructed a circSPEF2 overexpression model in EPC cells, alongside an empty vector control (pcDNA3.1), with three biological replicates in each group, followed by transcriptome sequencing. The efficiency of circSPEF2 overexpression in EPC cells was confirmed by qRT-PCR (Figure 4A). Principal component analysis (PCA) revealed that PC1 (39.33%) and PC2 (16.79%) could clearly distinguish between the two groups of samples, indicating that the subsequent RNA-seq differential expression analysis was reliable (Figure 4B). Based on the expression detection threshold (TPM ≥ 1), we conducted a Venn analysis between the two groups, revealing 187 specifically expressed genes in the circSPEF2 overexpression group, 704 specifically expressed genes in the control group, and 13,877 genes expressed in both groups (Figure 4C). To minimize interference from low-abundance transcripts and background noise, we focused the subsequent differential expression analysis on the 13,877 common genes. The differential expression analysis was performed using DESeq2, with the screening thresholds set as *p* < 0.05 and |log_2_FC|> 1. A total of 115 significantly differentially expressed genes (DEGs) were identified, with 45 genes upregulated and 70 downregulated (Figure 4D). The volcano plot visualizes the distribution of differentially expressed genes between the two groups, providing a reliable candidate set for downstream functional annotation and pathway enrichment analysis (Figure 4E). Heatmap clustering of differentially expressed genes showed significant differential expression in several genes related to sperm formation and maturation (Figure 4F). Among these, *spef2* encodes sperm-shaft-helix protein 2, which is highly expressed during the late stage of sperm cell metamorphosis and participates in the assembly of the sperm tail structure. *Prdm1a* inhibits stem cell genes and initiates meiosis, enabling spermatogonia to differentiate into sperm. The upregulation of these two genes correlates with the progression of sperm formation. In contrast, *zbtb16b* is gradually downregulated as spermatogonial stem cells enter the differentiation stage. During the nuclear concentration stage of later spermatogenesis, histone family proteins are replaced by protamine, leading to their gradual downregulation. Two glycosyltransferases, *galntl6* and *mgat3b*, are actively downregulated in the later stages of spermiogenesis to complete deglycosylation and expose the fertilization site. The downregulation of *cpeb3* expression in late spermatogenesis alleviates translational repression and initiates the translation of mRNAs, including those for cytoskeletal proteins and flagellar components, driving meiosis and sperm metamorphosis. The changes observed in these genes following circSPEF2 overexpression are consistent with the expression patterns of genes associated with late-stage sperm formation. Given that circSPEF2 is highly expressed in mature crucian carp testes, these findings suggest that circSPEF2 may be involved in regulating the expression of genes related to sperm formation and maturation. A GO annotation analysis of the DEGs indicated that these genes, primarily circSPEF2, focused on molecular functions related to binding and catalytic activities, cellular components associated with cellular anatomy, and biological processes relevant to cellular processes and biological regulation (Figure 4G). These results suggest that circSPEF2 may be involved in regulating genes associated with molecular binding and catalytic activities, which are potentially relevant to sperm tail assembly and function. However, further validation in germ cell models is required to confirm these possibilities. Additionally, it may serve as a key regulator of male germ cell differentiation and maturation. A KEGG enrichment analysis demonstrated that circSPEF2 overexpression significantly disrupted oocyte meiosis, ATP-dependent chromatin remodeling, and apoptosis while concomitantly dysregulating the mTOR, MAPK, Wnt, and cytokine–cytokine receptor interaction pathways (Figure 4H). Collectively, these pathways regulate sperm tail assembly, dynamic cytoskeleton remodeling, energy metabolism supply, and precise control of cell quality. These transcriptome results suggest that circSPEF2 may influence the expression of genes associated with sperm tail structure, including its parental gene *spef2*, and that it may participate in the regulatory network of spermatogenesis-related genes. However, direct evidence in germ cells is required to fully understand its role in spermatogenesis and maturation.

### 3.5. Verification of Screened Genes Using qRT-PCR

**Figure 5:**
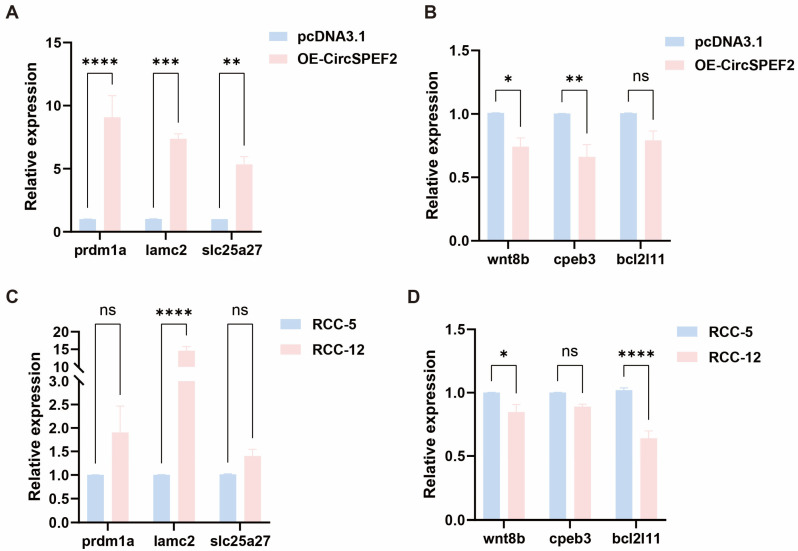
QRT-PCR verification of screened genes. (**A**,**B**) Relative expression levels of the six candidate genes in EPC cells transfected with the overexpression plasmid (oe-circSPEF2) or the control vector (pcDNA3.1). (**C**,**D**) Relative expression levels of six candidate genes in RCC testicular tissues with immature (RCC-5) and fully mature (RCC-12) gonads. * *p* < 0.05, ** *p* < 0.01, *** *p* < 0.001, **** *p* < 0.0001; ns, not significant.

To confirm the transcriptome data, we identified three up-regulated and three down-regulated genes related to gonadal development and verified their expression using qRT-PCR. At the cellular level, the circSPEF2 overexpression group exhibited significantly higher expression levels of *prdm1a*, *lamc2*, and *slc25a27* than the pcDNA3.1 control group (Figure 5A) and significantly lower expression levels of *wnt8b*, *cpeb3*, and *bcl2l11* (Figure 5B). Similarly, the expression of *prdm1a*, *lamc2*, and *slc25a27* was elevated in mature testes (Figure 5C). These findings suggest a relationship with the mature state of the testis and may indicate roles in maintaining the differentiation of late spermatogenic cells, strengthening the basement membrane structure of spermatogenic tubules, and enhancing the energy metabolism of sperm mitochondria, respectively. Conversely, the expression of *wnt8b*, *cpeb3*, and *bcl2l11* decreased in the mature testes (Figure 5D), reflecting the completion of early gonadal differentiation and the stabilization of spermatogenesis. These results indicate that the expression of circSPEF2 is closely linked to the expression patterns of key genes involved in gonadal development, highlighting its significant regulatory role in the testicular development of fish.

### 3.6. siRNA-Mediated Knockdown of circSPEF2 Alters the Expression of Six Candidate Genes

**Figure 6:**
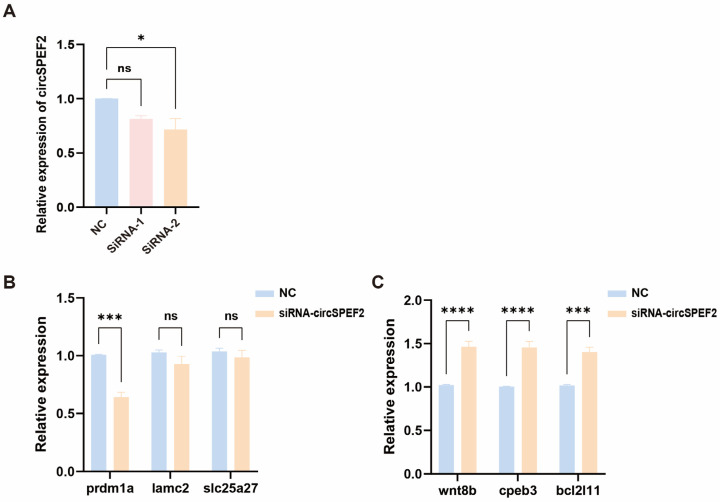
Knockdown of circSPEF2 and its effects on candidate gene expression in EPC cells. (**A**) Knockdown efficiency of circSPEF2 in EPC cells using siRNA-1 and siRNA-2. (**B**) QRT-PCR validation of three upregulated genes (*prdm1a*, *lamc2*, and *slc25a27*) following circSPEF2 knockdown. (**C**) QRT-PCR validation of three downregulated genes (*wnt8b*, *cpeb3*, and *bcl2l11*) following circSPEF2 knockdown. * *p* < 0.05, *** *p* < 0.001, **** *p* < 0.0001; ns, not significant.

To further verify the regulatory effect of circSPEF2 on the expression of candidate genes, we transfected siRNA targeting circSPEF2 into EPC cells to achieve its specific knockdown. The qRT-PCR results showed that the knockdown efficiency of siRNA-2 was the most significant compared with the control group (Figure 6A). Therefore, in subsequent experiments, siRNA-2 was used for interference treatment. Next, we further detected the expression levels of the six candidate differentially expressed genes selected using transcriptome sequencing. The results indicated that the three genes that were upregulated in the circSPEF2 overexpression group were significantly downregulated after knockdown (Figure 6B), while the three genes that were originally downregulated were significantly upregulated after knockdown (Figure 6C). The overall change trend was opposite to that of the overexpression result. The above results further indicate that circSPEF2 may participate in regulating the expression of these candidate genes, and in male fish, they may play a role in the development process.

### 3.7. Screening of Potential Binding Proteins of circSPEF2

**Figure 7:**
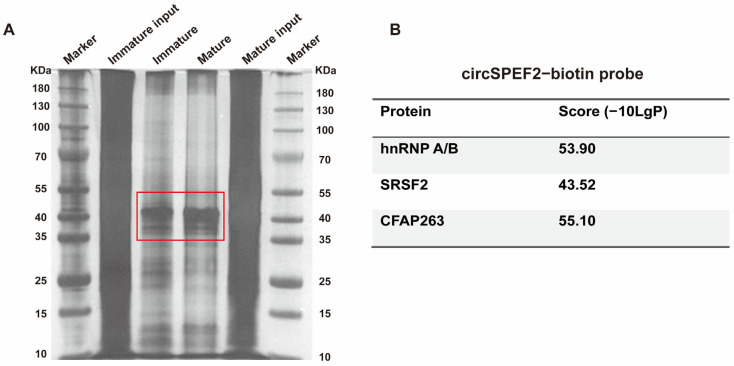
Identification of circSPEF2-binding proteins by RNA pull-down. (**A**) Proteins pulled down by the biotin-labeled circSPEF2 probe from immature and mature crucian carp testes were resolved using SDS-PAGE and visualized using silver staining. Input represents total protein lysates before pull-down. The red box marks the excised region (35–55 kDa) for LC-MS/MS analysis. (**B**) List of three candidate binding proteins identified by LC-MS/MS with their corresponding scores (−10LgP).

To identify potential RNA-binding proteins of circSPEF2, the total proteins extracted from immature and mature RCC gonadal tissues were subjected to RNA pull-down using a biotin-labeled circSPEF2 probe, followed by silver staining. Fewer protein bands were pulled down from mature tissues than from immature tissues (Figure 7A), consistent with the extensive functional protein degradation characteristic of late spermiogenesis. Given that most RNA-binding proteins are greater than 35 kDa and our silver-stained protein gel showed the highest band density and abundance within the 35–55 kDa range, we excised the differential bands in this region for an LC-MS/MS analysis. Mass spectrometry data were filtered using criteria of −10LgP ≥ 20, Unique ≥ 1, Peptides ≥ 2, and Coverage ≥ 5% to remove low-confidence identifications. A complete list of identified proteins is provided in Table S2. Protein expression levels were compared between mature and immature samples to identify proteins that bind specifically in the mature stage. Following gene annotation-based exclusion of non-specific circRNA-binding proteins such as histones, ribosomal proteins, cytoskeletal proteins, and abundant metabolic enzymes, three candidate proteins were identified: heterogeneous nuclear ribonucleoprotein A/B (hnRNP A/B), serine/arginine-rich splicing factor 2 (SRSF2), and cilia- and flagella-associated protein 263 (CFAP263). These proteins are listed in Figure 7B.

Given the roles of hnRNP A/B in mRNA stability, export, and translation, and those of SRSF2 in alternative splicing, we propose that cytoplasmic circSPEF2 binds to hnRNP A/B to modulate its localization and RNA-binding activity. This interaction may promote the translation of maturation genes, including *prdm1a*, *lamc2*, and *slc25a27*, and suppress the post-transcriptional maintenance of proliferation or apoptosis genes, such as *wnt8b*, *cpeb3*, and *bcl2l11*. A small fraction of circSPEF2 localized within the nucleus may affect the activity of splicing factors such as SRSF2, thereby helping to fine-tune the alternative splicing patterns of genes related to gonadal maturation. Notably, CFAP263 (also known as CCDC113, Coiled-Coil Domain Containing 113), another candidate binding protein identified in our screen, serves as a critical structural component of the spermatozoon neck. Its deficiency is known to cause the ace-phalic spermatozoa phenotype in humans and mice [26]. Furthermore, mutations in the family protein CFAP206 have been shown to cause severe abnormalities in sperm flagella [27]. As the parental gene of circSPEF2, *spef2* mediates Golgi-associated transport in male germ cells. Leveraging this functional context, we hypothesize that circSPEF2 post-transcriptionally governs CFAP263 expression and isoform specification, ensuring proper assembly of the basal body–neck transition zone to synergistically drive sperm tail formation and functional maturation.

## 4. Discussion

In mammals, gonadogenesis begins with the formation of the genital ridge, followed by differentiation into either testes or ovaries, a process orchestrated by the spatiotemporal expression of sex-determining genes and precise hormonal cues [28]. In contrast, sex differentiation in many fish and reptiles exhibits greater plasticity, with more complex influencing and regulatory mechanisms. Research on gonadal development and reproductive mechanisms in aquatic animals has mainly focused on the functional characterization of key sex differentiation genes, including representative genes involved in the male and female pathways, such as *dmy*, *sdy*, *dmrt1*, *amh*, *sox9*, *foxl2*, *wnt4*, and *rspo1* [1]. However, a growing body of evidence indicates that sex determination and gonadal differentiation are governed not only by the coding sequences of these key genes but also by their tightly orchestrated spatiotemporal expression patterns, shaped by multi-layered epigenetic control [29]. These epigenetic mechanisms mainly include DNA methylation, covalent histone modifications, and particularly gene silencing mediated by non-coding RNAs [30,31].

A growing body of circRNA expression profiles has been extensively utilized to analyze both the gonadal development process and pathological gonadal state [32,33]. High-throughput profiling in zebrafish, Nile tilapia, carp, golden trevally, and other teleost species has revealed a diverse array of circRNAs differentially expressed between mature testes and ovaries. The number and identity of these circRNAs exhibit significant sex specificity, suggesting a potential role in gonadal development or maintenance [19,34,35,36]. In this context, this study focused on the male gonad of crucian carp, identifying a circSPEF2, which is specifically and highly expressed in the mature testis. Its function aligns with that of circRNAs reported in other species, as they are involved in the fine regulation of germ cell development. For example, circSRY is specifically expressed in mouse testes; circINHA is specifically expressed in pig ovaries; and functional circRNAs have been documented in various fish species, including medaka, tongue sole, and rice field eel [22,23,37,38,39,40]. This study presents the systematic functional dissection of circSPEF2 in the mature testis of crucian carp.

CircSPEF2 was confirmed as a genuine circRNA, exhibiting significantly elevated expression in mature (12-month-old) testes of red crucian carp. QRT-PCR analysis across eight tissues and three developmental stages demonstrated testis-specific expression that progressively increased with testicular maturation, underscoring its role in male gonadal development. Subcellular localization studies indicated cytoplasmic enrichment. However, luciferase reporter assays suggested a low likelihood of ceRNA-based regulation. Nevertheless, functional studies were conducted, and a transcriptome analysis following circSPEF2 overexpression identified 45 upregulated and 70 down-regulated DEGs. GO and KEGG pathway analyses indicated that circSPEF2 significantly affects the expression of genes and pathways related to gonadal development. Functional validation via siRNA-mediated knockdown and qRT-PCR revealed opposite expression trends: genes that were upregulated upon circSPEF2 overexpression were downregulated following knockdown, while downregulated genes were upregulated. Together, these results demonstrate that circSPEF2 regulates these candidate genes. Subsequently, three candidate circSPEF2-binding proteins, designated hnRNP A/B, SRSF2, and CFAP263, were successfully identified through RNA pull-down followed by mass spectrometry. These findings provide crucial clues for deciphering the regulatory mechanism of circSPEF2 in male gonadal development in crucian carp. Future studies should focus on characterizing the specific interactions between circSPEF2 and these candidate proteins, as well as their downstream regulatory networks, to further elucidate the important role of circSPEF2-mediated post-transcriptional regulation in vertebrate male reproductive development.

## 5. Conclusions

This study demonstrates for the first time the regulatory role of circSPEF2 in male gonadal development in carp through expression profiling across developmental stages, functional validation, and RNA pull-down assays. Three candidate binding proteins were identified, namely, hnRNP A/B, SRSF2, and CFAP263. These findings provide new insights into the molecular mechanisms by which circRNAs regulate fish reproductive development. Future research should further explore the interaction mechanisms between circSPEF2 and these candidate proteins, as well as their downstream target pathways, thereby offering potential targets for circRNA-based interventions to improve fish gonadal development. Moreover, this discovery is expected to provide a novel perspective for studying the regulatory mechanisms of male reproductive development in vertebrates and holds significant reference value for fish genetic breeding, as well as mammalian reproductive biology research.

## Data Availability

The original contributions presented in this study are included in the article. Further inquiries can be directed to the corresponding authors. The raw RNA-seq data have been deposited in the NCBI Sequence Read Archive (SRA) under accession number PRJNA1434706.

## Supplementary Materials

The following supporting information can be downloaded at https://www.mdpi.com/article/10.3390/biology15090669/s1: Table S1. Sequences of primers and siRNAs used in this study; Table S2. Candidate RNA-binding proteins from mass spectrometry at mature and immature stages.

## Abbreviations

The following abbreviations are used in this manuscript:

SPEF2	sperm flagellar axonemal protein 2
RCC	red crucian carp
WCC	white crucian carp
WR	WCC ♀ × RCC ♂
WR-II	WR ♀ × WCC ♂
MMAF EPC	Multiple Morphological Abnormalities of the Flagella Epithelioma Papulosum Cyprini
PBS	Phosphate-buffered saline
DMEM	Dulbecco’s Modified Eagle Medium
FBS	Fetal Bovine Serum
FISH	Fluorescence In Situ Hybridization
ceRNA	competing endogenous RNA
PCA	Pareto-scaled principal component analysis
GO	Gene Ontology
KEGG	Kyoto Encyclopedia of Genes and Genomes
