# Supplementary material for: A Novel Circular RNA circSPEF2 Regulates Testis Development in Crucian Carp
Source: Biology (Basel). 2026 Apr 23;15(9):669. doi: 10.3390/biology15090669 (PMC13162906; doi:10.3390/biology15090669)
Supplement: Supplementary file 1 [file biology-15-00669-s001.zip › Table S1. Sequences of primers and siRNAs used in this study.pdf]

Table S1. Sequences of primers and siRNAs used in this study.

Primer or gene name	Sequence（5'→3'）	Annealing temperature	Product length	Purpose
**circSPEF2**	F: GGAGAGGGATGGTTCTCCAA<br>R: CTCCTGCATCAGCTTCACTG	60.12	120	PCR, qRT-PCR, circular validation, gene quantification, cell localization
spef2	F: AAATCAGCAACGCTCTTGGT<br>R: TCACAGCCTGTTTTTCCTGC	59.36	120	quantification of genes
prdm1a	F: ATTCTACCCTAACCCGCACT<br>R: CCTCAGACGGAAGCATACC	56.57	167	qRT-PCR
lamc2	F: CCTATCTGGGGAATCAAGCG<br>R: GGAACCACGGTCCTCAAGTC	56.95	157	qRT-PCR
slc25a27	F: TCCTCTGGACCTTACCAAAACC<br>R: CCCTCTTCTCGCACTATACCC	57.92	132	qRT-PCR
wnt8b	F: GTGGTCAGGGATGGCTATGG<br>R: CGTCCTACTTCGTTGTTGTGC	57.94	139	qRT-PCR
cpeb3	F: CACCAGACGCTCATCAATAAAGT<br>R: GATGGGGTTGAGGTGAGACG	56.79	159	qRT-PCR
bcl2l11	F: TGCCAAGTTCCCCGCTAA<br>R: CGTCCAATAAGGTCATTCATCC	55.86	262	qRT-PCR
β-actin	F: GATGAGATTGGCATGGCTTT<br>R: CACCTTCACCGTTCCAGTTT	59.36	122	internal reference genes
**siRNA-1**	S: AGAAUCAGUCUGAUACCAGTT<br>AS: CUGGUAUCAGACUGAUUUCUTT	N/A	N/A	siRNA-mediated knockdown
**siRNA-2**	S: CUAGAAUCAGUCUGAUAACCTT<br>AS: GGUAUCAGACUGAUUCUAGTT	N/A	N/A	siRNA-mediated knockdown

Note: For siRNA sequences, annealing temperature and product length are not applicable (N/A) as they are not used for PCR amplification. The sense and antisense strands are listed as RNA sequences (with U instead of T).
